# 1-[(*E*)-2-(5-*tert*-Butyl-2-hy­droxy­phen­yl)diazen-1-yl]naphthalen-2-ol

**DOI:** 10.1107/S1600536814001731

**Published:** 2014-01-29

**Authors:** Hassiba Bougueria, Assia Mili, Ali Benosmane, Abd el kader Bouchoul, Salaheddine Bouaoud

**Affiliations:** a Unité de Recherche de Chimie de l’Environnement et Moléculaire Structurale (CHEMS), Département de Chimie, Université Mentouri de Constantine 1, 25000 Constantine, Algeria

## Abstract

The non-H atoms of the title compound, C_20_H_20_N_2_O_2_, is located on a mirror plane except two methyl groups of the *tert*-butyl group. Intra­molecular N—H⋯O hydrogen bonds exist between the hy­droxy and diazenyl groups. In the crystal, mol­ecules are linked by weak C—H⋯O hydrogen bonds into supra­molecular chains running along the *a*-axis direction.

## Related literature   

For general background to azo compounds and their use in dyes, pigments and advanced materials, see: Lee *et al.* (2004 ▶). For related azo compounds, see: Yazıcı *et al.* (2010 ▶); Karadayı *et al.* (2006 ▶); Oakes (2002 ▶); Olivieri *et al.* (1989 ▶). For the synthesis, see: Wang *et al.* (2003 ▶).
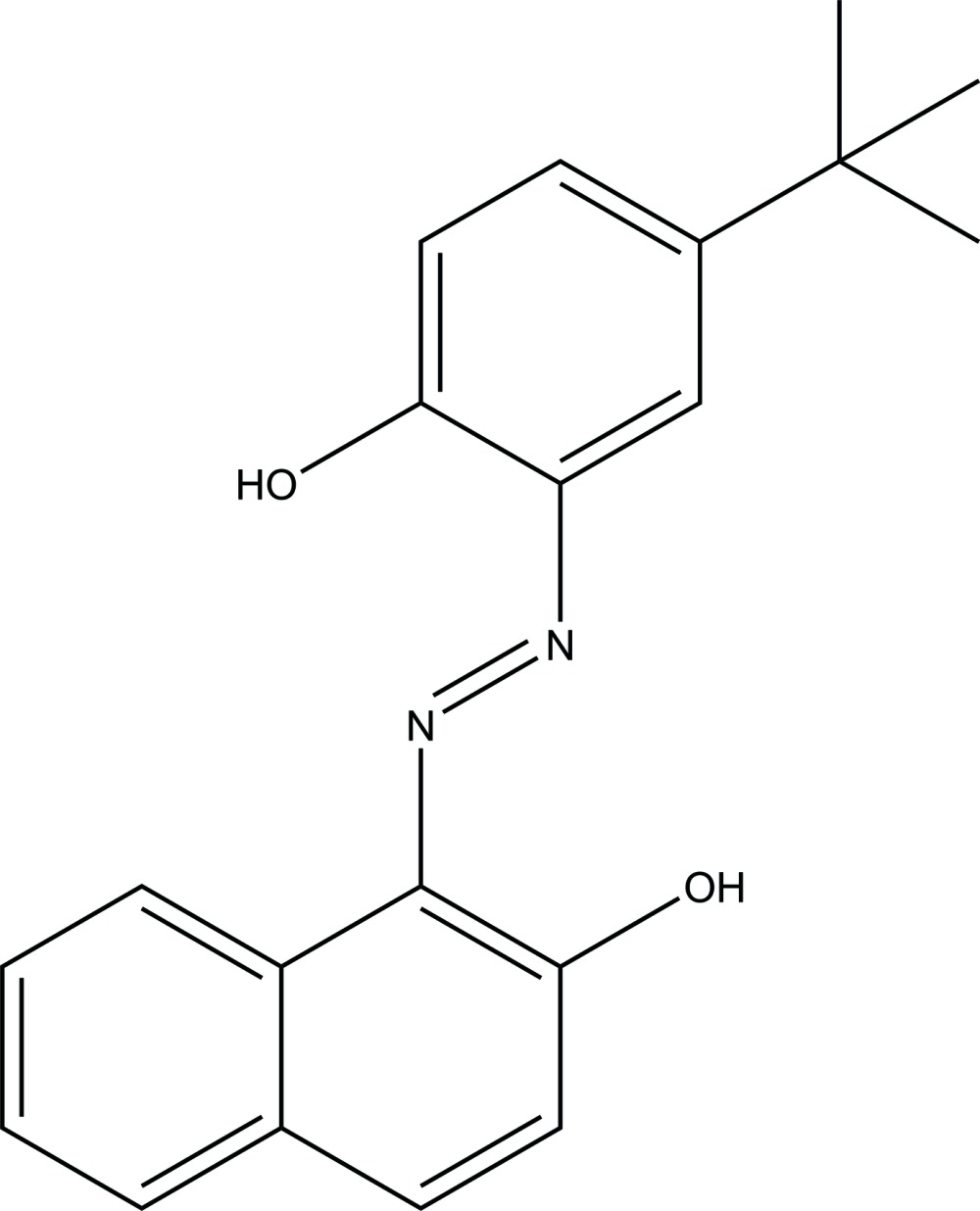


## Experimental   

### 

#### Crystal data  

C_20_H_20_N_2_O_2_
*M* *_r_* = 320.38
Monoclinic, 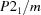

*a* = 9.696 (5) Å
*b* = 6.606 (5) Å
*c* = 13.385 (5) Å
β = 110.249 (5)°
*V* = 804.3 (8) Å^3^
*Z* = 2
Mo *K*α radiation
μ = 0.09 mm^−1^
*T* = 293 K
0.55 × 0.22 × 0.11 mm

#### Data collection  

Bruker APEXII diffractometer
Absorption correction: multi-scan (*SADABS*; Sheldrick, 2002 ▶) *T*_min_ = 0.978, *T*_max_ = 0.991
8889 measured reflections
2642 independent reflections
1767 reflections with *I* > 2σ(*I*)
*R*_int_ = 0.025

#### Refinement  

*R*[*F* ^2^ > 2σ(*F* ^2^)] = 0.048
*wR*(*F* ^2^) = 0.144
*S* = 0.89
2642 reflections
142 parameters
12 restraints
H-atom parameters constrained
Δρ_max_ = 0.43 e Å^−3^
Δρ_min_ = −0.23 e Å^−3^

### 

Data collection: *APEX2* (Bruker, 2006 ▶); cell refinement: *SAINT* (Bruker, 2006 ▶); data reduction: *SAINT*; program(s) used to solve structure: *SIR97* (Altomare *et al.*, 1999 ▶); program(s) used to refine structure: *SHELXL97* (Sheldrick, 2008 ▶); molecular graphics: *ORTEP-3 for Windows* (Farrugia, 2012 ▶); software used to prepare material for publication: *WinGX* (Farrugia, 2012 ▶).

## Supplementary Material

Crystal structure: contains datablock(s) global, I. DOI: 10.1107/S1600536814001731/xu5764sup1.cif

Structure factors: contains datablock(s) I. DOI: 10.1107/S1600536814001731/xu5764Isup2.hkl

Supporting information file. DOI: 10.1107/S1600536814001731/xu5764Isup3.cml

CCDC reference:

Additional supporting information:  crystallographic information; 3D view; checkCIF report

## Figures and Tables

**Table 1:** Hydrogen-bond geometry (Å, °)

*D*—H⋯*A*	*D*—H	H⋯*A*	*D*⋯*A*	*D*—H⋯*A*
O1—H1⋯N2	0.82	1.83	2.538 (3)	143
O2—H2⋯N1	0.82	1.93	2.630 (3)	142
C9—H9⋯O1^i^	0.93	2.52	3.338 (4)	146

Symmetry code: (i) 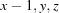.

**Fig. 1.**
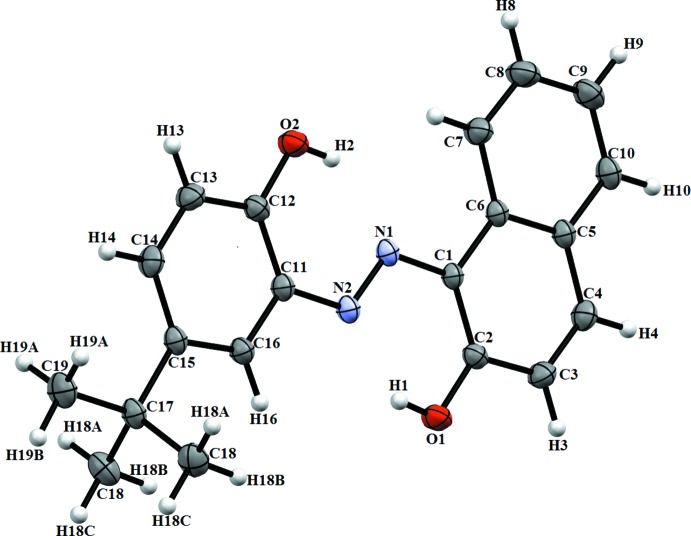
The molecular structure of the title compound, displacement ellipsoids are drawn at 50% probability level.

## supplementary crystallographic information

### 1. Comment

Azo compounds are very important in the fields of dyes, pigments and advanced materials (Lee *et al.*, 2004). Azo dyes are synthetic colours that contain an azo group, as part of the structure. We are involved in the color generation mechanism of azo pigments typically characterized by the chromophore of the azo group (–N=N–). However, some types of azo pigments are also known to possess the hydrazone structure (=N–NH–), often leading to the formation of intramolecular hydrogen bonds. The azo– hydrazone tautomerism in azo dyes has been known for more than a hundred years and is directly connected with the presence of at least one protic donor group in conjugation to the azo bridge (i.e. 2-naphthol) (Olivieri *et al.*., 1989). In particular, azo dyes that contain a naphtholic hydroxy group conjugated with the azo linkage exist in aqueous solution as an equilibrium mixture of two chemically distinct tautomers, the azo or hydrazone forms (Oakes, 2002). It is suggested that in a real azo compound the N=N double bond should have a length of 1.20–1.28 Å and the bond length of N–N single bonds, as in hydrazone tautomers, should be more than 1.4 Å. In the title compound, N–N bond lengths are 1.287 Å for N1–N2 , between the suggested N=N double bond and N–N single bond lengths. In the molecule, all bond lengths are in good agreement with those reported for other azo compounds (Yazıcı *et al.*, 2010; Karadayı *et al.*, 2006). We report here in the crystal structure of the title compound, obtained through the diazotization of 4-*tert*-butyl-2-hydroxy aniline followed by a coupling reaction with 2-naphthol.

The molecule of the title compound, with the atom numbering scheme, is shown in Fig. 1, crystallizes in the monoclinic space group P21/m. The molecular structure C_20_H_20_N_2_O_2_ is shown in Figure 1 The molecule adopts an anti–configuration with the two aryl groups reside on the opposite side of azo–group. The intramolecular N—H···O hydrogen bond is found (Table 1). In the crystal molecules are linked by the weak C—H···O interactions into chains.

### 2. Experimental

The title compound was obtained through the diazotization of 4-*tert*-butyl-2-hydroxyaniline followed by a coupling reaction with 2-naphthol, according to the literature procedure used to synthesize other aromatic azo-compounds (Wang *et al.*, 2003). Single crystals of the title compound were obtained by slow evaporation at room temperature of a solution in DMSO.

### 3. Refinement

H atoms, attached to carbon atoms have been placed in geometrically idealized positions and refined as riding, with C—H = 0.93 (aromatic) and 0.96 Å (methyl), and U_iso_(H) = 1.2U_eq_(C) or 1.5U_eq_(methyl C). hydroxy H atoms were introduced in calculated positions and treated as riding on their parent atoms with O—H = 0.82 Å and U_iso_(H) = 1.5U_eq_(O).

### Crystal data

**Table:**

C_20_H_20_N_2_O_2_	*F*(000) = 340
*M**_r_* = 320.38	*D*_x_ = 1.323 Mg m^−^^3^
Monoclinic, *P*2_1_/*m*	Mo *K*α radiation, λ = 0.71073 Å
Hall symbol: -P 2yb	Cell parameters from 2015 reflections
*a* = 9.696 (5) Å	θ = 3.2–30.4°
*b* = 6.606 (5) Å	µ = 0.09 mm^−^^1^
*c* = 13.385 (5) Å	*T* = 293 K
β = 110.249 (5)°	Prism, red
*V* = 804.3 (8) Å^3^	0.55 × 0.22 × 0.11 mm
*Z* = 2	

### Data collection

**Table:**

Bruker APEXII diffractometer	2642 independent reflections
Radiation source: fine-focus sealed tube	1767 reflections with *I* > 2σ(*I*)
Graphite monochromator	*R*_int_ = 0.025
CCD rotation images, thin slices scans	θ_max_ = 30.5°, θ_min_ = 3.2°
Absorption correction: multi-scan (*SADABS*; Sheldrick, 2002)	*h* = −13→13
*T*_min_ = 0.978, *T*_max_ = 0.991	*k* = −7→9
8889 measured reflections	*l* = −18→19

### Refinement

**Table:**

Refinement on *F*^2^	Primary atom site location: structure-invariant direct methods
Least-squares matrix: full	Secondary atom site location: difference Fourier map
*R*[*F*^2^ > 2σ(*F*^2^)] = 0.048	Hydrogen site location: inferred from neighbouring sites
*wR*(*F*^2^) = 0.144	H-atom parameters constrained
*S* = 0.89	*w* = 1/[σ^2^(*F*_o_^2^) + (0.0766*P*)^2^ + 0.359*P*] where *P* = (*F*_o_^2^ + 2*F*_c_^2^)/3
2642 reflections	(Δ/σ)_max_ < 0.001
142 parameters	Δρ_max_ = 0.43 e Å^−^^3^
12 restraints	Δρ_min_ = −0.23 e Å^−^^3^

### Special details

**Table:**

Geometry. Bond distances, angles etc. have been calculated using the rounded fractional coordinates. All su's are estimated from the variances of the (full) variance-covariance matrix. The cell esds are taken into account in the estimation of distances, angles and torsion angles
Refinement. Refinement on F^2^ for ALL reflections except those flagged by the user for potential systematic errors. Weighted R-factors wR and all goodnesses of fit S are based on F^2^, conventional R-factors R are based on F, with F set to zero for negative F^2^. The observed criterion of F^2^ > 2sigma(F^2^) is used only for calculating -R-factor-obs etc. and is not relevant to the choice of reflections for refinement. R-factors based on F^2^ are statistically about twice as large as those based on F, and R-factors based on ALL data will be even larger.

### Fractional atomic coordinates and isotropic or equivalent isotropic displacement parameters (Å^2^)

**Table:**

	*x*	*y*	*z*	*U*_iso_*/*U*_eq_	
O1	0.77215 (13)	0.75000	0.56321 (10)	0.0270 (4)	
O2	0.31274 (13)	0.75000	0.19510 (10)	0.0277 (4)	
N1	0.48377 (15)	0.75000	0.39638 (11)	0.0180 (4)	
N2	0.60014 (15)	0.75000	0.37081 (11)	0.0194 (4)	
C1	0.50711 (18)	0.75000	0.50553 (13)	0.0164 (4)	
C2	0.64717 (17)	0.75000	0.58490 (14)	0.0195 (4)	
C3	0.66094 (19)	0.75000	0.69373 (14)	0.0242 (5)	
C4	0.53890 (19)	0.75000	0.72205 (14)	0.0240 (5)	
C5	0.39521 (18)	0.75000	0.64529 (14)	0.0191 (5)	
C6	0.37768 (17)	0.75000	0.53520 (13)	0.0172 (4)	
C7	0.23305 (19)	0.75000	0.46054 (15)	0.0258 (5)	
C8	0.1138 (2)	0.75000	0.49354 (16)	0.0326 (6)	
C9	0.1312 (2)	0.75000	0.60179 (16)	0.0300 (6)	
C10	0.2695 (2)	0.75000	0.67614 (15)	0.0246 (5)	
C11	0.57989 (18)	0.75000	0.26191 (13)	0.0190 (4)	
C12	0.44281 (18)	0.75000	0.17815 (14)	0.0209 (5)	
C13	0.44203 (19)	0.75000	0.07401 (14)	0.0259 (5)	
C14	0.5727 (2)	0.75000	0.05311 (14)	0.0254 (5)	
C15	0.71035 (18)	0.75000	0.13444 (13)	0.0195 (5)	
C16	0.70929 (18)	0.75000	0.23809 (14)	0.0203 (5)	
C17	0.85759 (19)	0.75000	0.11508 (14)	0.0220 (5)	
C18	0.94634 (15)	0.5609 (2)	0.16604 (12)	0.0300 (4)	
C19	0.8368 (2)	0.75000	−0.00394 (16)	0.0327 (6)	
H1	0.75270	0.75000	0.49850	0.0410*	
H2	0.32790	0.75000	0.25930	0.0420*	
H3	0.75380	0.75000	0.74610	0.0290*	
H4	0.55030	0.75000	0.79390	0.0290*	
H7	0.21840	0.75000	0.38810	0.0310*	
H8	0.01970	0.75000	0.44290	0.0390*	
H9	0.04930	0.75000	0.62290	0.0360*	
H10	0.28130	0.75000	0.74810	0.0300*	
H13	0.35290	0.75000	0.01770	0.0310*	
H14	0.56850	0.75000	−0.01730	0.0310*	
H16	0.79880	0.75000	0.29410	0.0240*	
H18A	0.89250	0.44160	0.13420	0.0360*	
H18B	0.96370	0.56020	0.24120	0.0360*	
H18C	1.03870	0.56280	0.15450	0.0360*	
H19A	0.78450	0.63120	−0.03610	0.0390*	
H19B	0.93110	0.75000	−0.01250	0.0390*	

### Atomic displacement parameters (Å^2^)

**Table:**

	*U*^11^	*U*^22^	*U*^33^	*U*^12^	*U*^13^	*U*^23^
O1	0.0180 (6)	0.0410 (8)	0.0236 (6)	0.0000	0.0091 (5)	0.0000
O2	0.0191 (6)	0.0418 (8)	0.0233 (6)	0.0000	0.0088 (5)	0.0000
N1	0.0201 (6)	0.0172 (7)	0.0188 (7)	0.0000	0.0096 (5)	0.0000
N2	0.0208 (6)	0.0220 (7)	0.0178 (7)	0.0000	0.0096 (5)	0.0000
C1	0.0192 (7)	0.0150 (7)	0.0162 (7)	0.0000	0.0078 (6)	0.0000
C2	0.0174 (7)	0.0202 (8)	0.0219 (8)	0.0000	0.0082 (6)	0.0000
C3	0.0207 (8)	0.0303 (10)	0.0193 (8)	0.0000	0.0040 (6)	0.0000
C4	0.0272 (8)	0.0282 (10)	0.0169 (8)	0.0000	0.0080 (7)	0.0000
C5	0.0224 (8)	0.0173 (8)	0.0198 (8)	0.0000	0.0100 (6)	0.0000
C6	0.0201 (7)	0.0146 (8)	0.0185 (8)	0.0000	0.0086 (6)	0.0000
C7	0.0208 (8)	0.0371 (11)	0.0194 (8)	0.0000	0.0070 (7)	0.0000
C8	0.0188 (8)	0.0494 (13)	0.0298 (10)	0.0000	0.0085 (7)	0.0000
C9	0.0238 (8)	0.0384 (11)	0.0331 (10)	0.0000	0.0165 (8)	0.0000
C10	0.0298 (9)	0.0260 (9)	0.0232 (9)	0.0000	0.0159 (7)	0.0000
C11	0.0212 (7)	0.0197 (8)	0.0177 (8)	0.0000	0.0087 (6)	0.0000
C12	0.0191 (7)	0.0217 (9)	0.0233 (8)	0.0000	0.0093 (6)	0.0000
C13	0.0213 (8)	0.0357 (11)	0.0187 (8)	0.0000	0.0044 (6)	0.0000
C14	0.0296 (9)	0.0324 (10)	0.0160 (8)	0.0000	0.0102 (7)	0.0000
C15	0.0232 (8)	0.0183 (8)	0.0204 (8)	0.0000	0.0117 (7)	0.0000
C16	0.0196 (7)	0.0238 (9)	0.0193 (8)	0.0000	0.0089 (6)	0.0000
C17	0.0248 (8)	0.0242 (9)	0.0218 (8)	0.0000	0.0142 (7)	0.0000
C18	0.0306 (6)	0.0293 (7)	0.0356 (7)	0.0053 (6)	0.0185 (5)	0.0029 (6)
C19	0.0347 (10)	0.0448 (13)	0.0256 (9)	0.0000	0.0194 (8)	0.0000

### Geometric parameters (Å, º)

**Table:**

O1—C2	1.340 (2)	C14—C15	1.400 (3)
O2—C12	1.357 (3)	C15—C17	1.537 (3)
O1—H1	0.8200	C15—C16	1.391 (3)
O2—H2	0.8200	C17—C18^i^	1.536 (2)
N1—C1	1.399 (2)	C17—C18	1.536 (2)
N1—N2	1.288 (2)	C17—C19	1.535 (3)
N2—C11	1.402 (2)	C3—H3	0.9300
C1—C6	1.441 (3)	C4—H4	0.9300
C1—C2	1.405 (3)	C7—H7	0.9300
C2—C3	1.416 (3)	C8—H8	0.9300
C3—C4	1.362 (3)	C9—H9	0.9300
C4—C5	1.416 (3)	C10—H10	0.9300
C5—C6	1.424 (3)	C13—H13	0.9300
C5—C10	1.416 (3)	C14—H14	0.9300
C6—C7	1.413 (3)	C16—H16	0.9300
C7—C8	1.373 (3)	C18—H18A	0.9600
C8—C9	1.400 (3)	C18—H18B	0.9600
C9—C10	1.366 (3)	C18—H18C	0.9600
C11—C12	1.411 (3)	C19—H19A	0.9500
C11—C16	1.397 (3)	C19—H19B	0.9600
C12—C13	1.391 (3)	C19—H19A^i^	0.9500
C13—C14	1.389 (3)		
			
O1···N1	2.913 (3)	C11···C4^iii^	3.529 (3)
O1···N2	2.538 (3)	C11···C4^iv^	3.529 (3)
O1···C9^ii^	3.338 (4)	C11···C4^v^	3.529 (3)
O1···C7^iii^	3.317 (3)	C11···C5^iv^	3.508 (3)
O1···C7^iv^	3.317 (3)	C11···C5^v^	3.508 (3)
O1···C7^v^	3.317 (3)	C11···C10^iv^	3.591 (3)
O1···C7^vi^	3.317 (3)	C11···C10^v^	3.591 (3)
O1···C9^vii^	3.338 (4)	C11···C5^vi^	3.508 (3)
O2···N1	2.630 (3)	C11···C4^vi^	3.529 (3)
O2···N2	2.960 (3)	C11···C5^iii^	3.508 (3)
O1···H9^vii^	2.5200	C11···C10^iii^	3.591 (3)
O1···H9^ii^	2.5200	C11···C10^vi^	3.591 (3)
O2···H18C^viii^	2.8100	C12···C4^iii^	3.544 (3)
O2···H18C^ix^	2.8100	C12···C4^iv^	3.544 (3)
N1···O1	2.913 (3)	C12···C4^v^	3.544 (3)
N1···O2	2.630 (3)	C12···C4^vi^	3.544 (3)
N2···C5^iv^	3.311 (3)	C16···C10^iii^	3.480 (3)
N2···C5^v^	3.311 (3)	C16···C10^iv^	3.480 (3)
N2···C5^iii^	3.311 (3)	C16···C10^v^	3.480 (3)
N2···O1	2.538 (3)	C16···C10^vi^	3.480 (3)
N2···O2	2.960 (3)	C11···H1	3.0300
N2···C5^vi^	3.311 (3)	C14···H19A^i^	2.8200
N1···H2	1.9300	C14···H19A	2.8200
N1···H7	2.5400	C16···H18B	2.7500
N1···H1	2.4900	C16···H18B^i^	2.7500
N2···H1	1.8300	C18···H16	2.8700
N2···H2	2.5400	C19···H14	2.5500
C1···C1^iv^	3.307 (3)	H1···N1	2.4900
C1···C1^v^	3.307 (3)	H1···N2	1.8300
C1···C6^iv^	3.588 (3)	H1···C11	3.0300
C1···C6^v^	3.588 (3)	H2···N1	1.9300
C1···C1^iii^	3.307 (3)	H2···N2	2.5400
C1···C1^vi^	3.307 (3)	H2···H7	2.3200
C1···C6^iii^	3.588 (3)	H4···H10	2.4600
C1···C6^vi^	3.588 (3)	H4···H14^x^	2.4700
C4···C11^iv^	3.529 (3)	H4···H14^xi^	2.4700
C4···C11^v^	3.529 (3)	H7···N1	2.5400
C4···C12^iv^	3.544 (3)	H7···H2	2.3200
C4···C12^v^	3.544 (3)	H8···H16^viii^	2.3700
C4···C11^iii^	3.529 (3)	H8···H16^ix^	2.3700
C4···C11^vi^	3.529 (3)	H9···O1^viii^	2.5200
C4···C12^iii^	3.544 (3)	H9···O1^ix^	2.5200
C4···C12^vi^	3.544 (3)	H10···H4	2.4600
C5···N2^iv^	3.311 (3)	H14···C19	2.5500
C5···N2^v^	3.311 (3)	H14···H4^xii^	2.4700
C5···C11^iv^	3.508 (3)	H14···H19A	2.3300
C5···C11^v^	3.508 (3)	H14···H4^xiii^	2.4700
C5···N2^iii^	3.311 (3)	H14···H19A^i^	2.3300
C5···N2^vi^	3.311 (3)	H16···C18	2.8700
C5···C11^iii^	3.508 (3)	H16···H8^ii^	2.3700
C5···C11^vi^	3.508 (3)	H16···H18B	2.3300
C6···C1^iv^	3.588 (3)	H16···C18^i^	2.8700
C6···C1^v^	3.588 (3)	H16···H8^vii^	2.3700
C6···C1^iii^	3.588 (3)	H16···H18B^i^	2.3300
C6···C1^vi^	3.588 (3)	H18A···H19A	2.4900
C7···O1^iv^	3.317 (3)	H18A···H18A^xiv^	2.5300
C7···O1^v^	3.317 (3)	H18B···C16	2.7500
C7···O1^iii^	3.317 (3)	H18B···H16	2.3300
C7···O1^vi^	3.317 (3)	H18B···H18B^i^	2.5100
C9···O1^viii^	3.338 (4)	H18C···O2^ii^	2.8100
C9···O1^ix^	3.338 (4)	H18C···H19B	2.4500
C10···C11^iv^	3.591 (3)	H18C···O2^vii^	2.8100
C10···C11^v^	3.591 (3)	H18C···H18C^i^	2.4700
C10···C16^iv^	3.480 (3)	H19A···C14	2.8200
C10···C16^v^	3.480 (3)	H19A···H14	2.3300
C10···C11^iii^	3.591 (3)	H19A···H18A	2.4900
C10···C11^vi^	3.591 (3)	H19B···H18C	2.4500
C10···C16^iii^	3.480 (3)	H19B···H18C^i^	2.4500
C10···C16^vi^	3.480 (3)		
			
C2—O1—H1	110.00	C15—C17—C18^i^	109.60 (10)
C12—O2—H2	110.00	C18—C17—C18^i^	108.81 (14)
N2—N1—C1	115.96 (14)	C18^i^—C17—C19	108.24 (11)
N1—N2—C11	117.14 (14)	C18—C17—C19	108.24 (11)
N1—C1—C6	116.50 (15)	C15—C17—C18	109.60 (10)
N1—C1—C2	123.67 (16)	C2—C3—H3	120.00
C2—C1—C6	119.84 (15)	C4—C3—H3	120.00
O1—C2—C3	116.90 (16)	C3—C4—H4	119.00
O1—C2—C1	123.09 (16)	C5—C4—H4	119.00
C1—C2—C3	120.01 (16)	C6—C7—H7	120.00
C2—C3—C4	120.31 (17)	C8—C7—H7	120.00
C3—C4—C5	121.97 (16)	C7—C8—H8	119.00
C4—C5—C10	121.23 (16)	C9—C8—H8	119.00
C6—C5—C10	119.70 (16)	C8—C9—H9	120.00
C4—C5—C6	119.07 (16)	C10—C9—H9	120.00
C5—C6—C7	117.77 (16)	C5—C10—H10	119.00
C1—C6—C5	118.80 (15)	C9—C10—H10	120.00
C1—C6—C7	123.43 (15)	C12—C13—H13	120.00
C6—C7—C8	120.85 (17)	C14—C13—H13	120.00
C7—C8—C9	121.32 (19)	C13—C14—H14	119.00
C8—C9—C10	119.38 (19)	C15—C14—H14	119.00
C5—C10—C9	120.99 (18)	C11—C16—H16	119.00
C12—C11—C16	119.46 (16)	C15—C16—H16	118.00
N2—C11—C12	125.48 (16)	C17—C18—H18A	110.00
N2—C11—C16	115.06 (15)	C17—C18—H18B	110.00
C11—C12—C13	118.22 (17)	C17—C18—H18C	109.00
O2—C12—C13	118.99 (16)	H18A—C18—H18B	109.00
O2—C12—C11	122.79 (16)	H18A—C18—H18C	109.00
C12—C13—C14	120.85 (17)	H18B—C18—H18C	109.00
C13—C14—C15	122.27 (16)	C17—C19—H19A	109.00
C16—C15—C17	119.75 (15)	C17—C19—H19B	110.00
C14—C15—C16	116.19 (17)	C17—C19—H19A^i^	109.00
C14—C15—C17	124.06 (15)	H19A—C19—H19B	109.00
C11—C16—C15	123.01 (16)	H19A—C19—H19A^i^	111.00
C15—C17—C19	112.28 (15)	H19A^i^—C19—H19B	109.00
			
C1—N1—N2—C11	180.00 (1)	C6—C5—C10—C9	0.00 (1)
N2—N1—C1—C2	0.00 (1)	C1—C6—C7—C8	180.00 (1)
N2—N1—C1—C6	180.00 (1)	C5—C6—C7—C8	0.00 (1)
N1—N2—C11—C12	0.00 (1)	C6—C7—C8—C9	0.00 (1)
N1—N2—C11—C16	180.00 (1)	C7—C8—C9—C10	0.00 (1)
N1—C1—C2—O1	0.00 (1)	C8—C9—C10—C5	0.00 (1)
N1—C1—C2—C3	180.00 (1)	N2—C11—C12—O2	0.00 (1)
C6—C1—C2—O1	180.00 (1)	N2—C11—C12—C13	180.00 (1)
C6—C1—C2—C3	0.00 (1)	C16—C11—C12—O2	180.00 (1)
N1—C1—C6—C5	180.00 (1)	C16—C11—C12—C13	0.00 (1)
N1—C1—C6—C7	0.00 (1)	N2—C11—C16—C15	180.00 (1)
C2—C1—C6—C5	0.00 (1)	C12—C11—C16—C15	0.00 (1)
C2—C1—C6—C7	180.00 (1)	O2—C12—C13—C14	180.00 (1)
O1—C2—C3—C4	180.00 (1)	C11—C12—C13—C14	0.00 (1)
C1—C2—C3—C4	0.00 (1)	C12—C13—C14—C15	0.00 (1)
C2—C3—C4—C5	0.00 (1)	C13—C14—C15—C16	0.00 (1)
C3—C4—C5—C6	0.00 (1)	C13—C14—C15—C17	180.00 (1)
C3—C4—C5—C10	180.00 (1)	C14—C15—C16—C11	0.00 (1)
C4—C5—C6—C1	0.00 (1)	C17—C15—C16—C11	180.00 (1)
C4—C5—C6—C7	180.00 (1)	C14—C15—C17—C18	−120.32 (10)
C10—C5—C6—C1	180.00 (1)	C14—C15—C17—C19	0.00 (1)
C10—C5—C6—C7	0.00 (1)	C16—C15—C17—C18	59.68 (10)
C4—C5—C10—C9	180.00 (1)	C16—C15—C17—C19	180.00 (1)

Symmetry codes: (i) *x*, −*y*+3/2, *z*; (ii) *x*+1, *y*, *z*; (iii) −*x*+1, −*y*+1, −*z*+1; (iv) −*x*+1, *y*−1/2, −*z*+1; (v) −*x*+1, *y*+1/2, −*z*+1; (vi) −*x*+1, −*y*+2, −*z*+1; (vii) *x*+1, −*y*+3/2, *z*; (viii) *x*−1, *y*, *z*; (ix) *x*−1, −*y*+3/2, *z*; (x) *x*, *y*, *z*+1; (xi) *x*, −*y*+3/2, *z*+1; (xii) *x*, *y*, *z*−1; (xiii) *x*, −*y*+3/2, *z*−1; (xiv) *x*, −*y*+1/2, *z*.

### Hydrogen-bond geometry (Å, º)

**Table:**

*D*—H···*A*	*D*—H	H···*A*	*D*···*A*	*D*—H···*A*
O1—H1···N2	0.82	1.83	2.538 (3)	143
O2—H2···N1	0.82	1.93	2.630 (3)	142
C9—H9···O1^viii^	0.93	2.52	3.338 (4)	146

Symmetry code: (viii) *x*−1, *y*, *z*.
